# Dynamics of Weeds in the Soil Seed Bank: A Hidden Markov Model to Estimate Life History Traits from Standing Plant Time Series

**DOI:** 10.1371/journal.pone.0139278

**Published:** 2015-10-01

**Authors:** Benjamin Borgy, Xavier Reboud, Nathalie Peyrard, Régis Sabbadin, Sabrina Gaba

**Affiliations:** 1 INRA, UMR1347 Agroécologie, Dijon, France; 2 INRA, UR875 Unité de Mathématiques et Informatique Appliquées, Toulouse, France; 3 Centre National de Recherche Scientifique, Centre d'Ecologie Fonctionnelle et Evolutive, UMR 5175, Montpellier, France; California State University, Fresno, CA, UNITED STATES

## Abstract

Predicting the population dynamics of annual plants is a challenge due to their hidden seed banks in the field. However, such predictions are highly valuable for determining management strategies, specifically in agricultural landscapes. In agroecosystems, most weed seeds survive during unfavourable seasons and persist for several years in the seed bank. This causes difficulties in making accurate predictions of weed population dynamics and life history traits (LHT). Consequently, it is very difficult to identify management strategies that limit both weed populations and species diversity. In this article, we present a method of assessing weed population dynamics from both standing plant time series data and an unknown seed bank. We use a Hidden Markov Model (HMM) to obtain estimates of over 3,080 botanical records for three major LHT: seed survival in the soil, plant establishment (including post-emergence mortality), and seed production of 18 common weed species. Maximum likelihood and Bayesian approaches were complementarily used to estimate LHT values. The results showed that the LHT provided by the HMM enabled fairly accurate estimates of weed populations in different crops. There was a positive correlation between estimated germination rates and an index of the specialisation to the crop type (IndVal). The relationships between estimated LHTs and that between the estimated LHTs and the ecological characteristics of weeds provided insights into weed strategies. For example, a common strategy to cope with agricultural practices in several weeds was to produce less seeds and increase germination rates. This knowledge, especially of LHT for each type of crop, should provide valuable information for developing sustainable weed management strategies.

## Introduction

Agriculture has to face conflicting challenges such as ensuring food security and conserving biodiversity while reducing chemical inputs and environmental impacts and to conserve biodiversity [1]. In agroecosystems, weeds pose a major threat to crop production. Hence, weeds have been intensively managed over the decades resulting in a huge decline in weed biodiversity [2]. However, arable weeds also sustain many taxa in agroecosystems, including birds and pollinators [3–5] and one of the major challenges facing stakeholders is to predict the decline or increase in certain species in response to management actions. Therefore, there is a need to determine appropriate management strategies that maintain crop production while maintaining weed species biodiversity in agroecosystems.

Predicting the abundance of weed species and communities is a challenge because of the difficulty in characterization of the soil seed bank. Most weed species are therophytes [2,6] and survive as seeds during unfavourable seasons and eventually they complete their life cycle during more favourable seasons. The dormant seeds of many species can survive for years or decades until favourable conditions occur. The seed bank may contain several hundreds or thousands of seeds m^-2^. Counts as high as 50,000 seeds m^-2^ have been reported [7]. There is some consensus that 500 to 5,000 seeds m^-2^ is a median value of seed density m^-2^; although this range is still highly variable when pooled over all species that may be present in a field [8–10]. One way of describing weed soil seed bank is that they are primarily an assemblage of seeds that sometimes will germinate, emerge, and produce an adult plant. Unfortunately, little is known about the structure and composition of the persistent seed bank since counting and identifying seeds at the species level is tedious, expensive, and time-consuming. In addition, weed abundance are often only estimated by semi-quantitative measures derived from farmers’ perceptions of weed infestation [11]. While such perceptions are appropriate for describing highly variable situations within fields, they only provide a vague picture of the actual weed community and their variations over space and time. Furthermore, weed seed bank emergence may be spread over several growing seasons, thus, making it difficult to correlate weed populations with the weed management practices.

The objective of this paper is to estimate three life history traits of the most frequently occurring weed species in the field, i.e., seed survival, plant establishment and seed production. In arable fields, life history traits such as fecundity, establishment rate, seedling survival, and seed bank persistence have been identified as the most important in determining year-to-year changes in weed population or weed species occurrence [12, 13, 14, 15]. Weed establishment and species composition is highly dependent on the agricultural techniques used (e.g., tillage, crop type) and is influence by environmental variables (e.g., temperature, moisture and soil structure). Using model sensitivity analysis, Colbach et al. [13] confirmed that life history traits related to plant establishment and seed reproduction are key parameters in weed dynamics.

Hidden Markov Models (HMMs), which are classical extensions of Markov chains, are used to allow for missing (or hidden) data. HMM have already been used to estimate plant demographic parameters without seed bank observation in the case of feral populations of oilseed rape (*Brassica napus*) [16]. Recently, a study showed that the HMM offers a reliable way to test for the existence of a one-year seed bank on the sole basis of time series of patch occupancy data in metapopulations [17]. Here, the HMM approach is used at the field scale to estimate three major life history traits (LHTs), i.e., seed survival, plant establishment, and seed production, from time series of above-ground plant abundance data for the most common weed species under several management systems.

We first evaluate the approach by comparing the relationship between estimated LHTs and certain weed features (specialisation index, i.e., IndVal and functional traits) to current knowledge in ecology. Then, using the estimated LHTs to compute species growth rates, we compare weed life history strategies, which are synthetic characteristics that determine species population dynamics [18].

## Materials and Methods

### Dataset

A total of 3,080 weed records in 26 types of crop were obtained from 385 fields in France (latitudinal range: 761 km; longitudinal range: 696 km) between 2002 and 2009 as part of the national Biovigilance-Flore project [19] (S1 Fig). Each field was surveyed for up to eight successive years by two or more experts walking across the survey area (2000 m²) for a minimum of 20 min and recording the abundances of all weed species. Surveys took place in spring (between the end of March and the beginning of April) for winter-sown crops, and in summer (around the beginning of July) for spring- and summer-sown crops. This survey was generally made after herbicide treatments. The abundance scale adapted from Barralis [11] gives a semi-quantitative count of the number of individual weeds per m², ‘1’ indicates that no plant was found in the 2000 m² area; ‘2’ that one to two individual weeds were recorded per m²; ‘3’ that three to 20 individual weeds were recorded per m^2^; and ‘4’ that more than 20 individual weeds were recorded per m². Since this last abundance class does not have an upper bound and has a wide range of variation, additional abundance classes were created for the number of seeds in the seed bank (*c*_*y*_), resulting in six abundance classes: ‘1’ = 0 ind/m²; ‘2’ = {1:2} ind/m²; ‘3’ = {3:20} ind/m²; ‘4’ = {21:60} ind/m²; ‘5’ = {61:100} ind/m²; and ‘6’ = {101:+∞} ind/m². This made it possible to model the fact that not all the seeds in the seed bank may emerge and that a large number of emerged plants could either result from a high germination rate of a moderate size seed bank or a low germination rate of a large size seed bank.

Four management actions were selected corresponding to four common crop species and their associated agricultural practices—winter cereals, oilseed rape, maize (*Zea mays*) and sunflower (*Helianthus annuus L*.)—that were sufficiently representative of the dataset. We selected the fields (1) where at least one of these crops had been sown at least once, and (2) that had been surveyed for two consecutive years. This gave a total of 329 out of the 385 surveyed fields (i.e., time series) corresponding to 1,191 records. The average duration of a time series was 3.62 years (s.d. = 1.19 years). Management actions were unequally represented in the surveys (49.6% of winter cereals (WC), 10.2% of oilseed rape (OR), 29.3% of maize (M) and 10.8% of sunflower (SF)) for the whole dataset. Up to 288 different species were recorded. We focused on the annual weed species recorded in at least 120 surveys. We initially selected 32 weed species (S1 Table). Then, in order to improve the quality of our estimates, we removed the species for which the pairs (species, management action) were present in less than 10% of the total number of crop sequences recorded. Finally, we selected 18 weed species.

### Hidden Markov Model (HMM)

**Fig 1:**
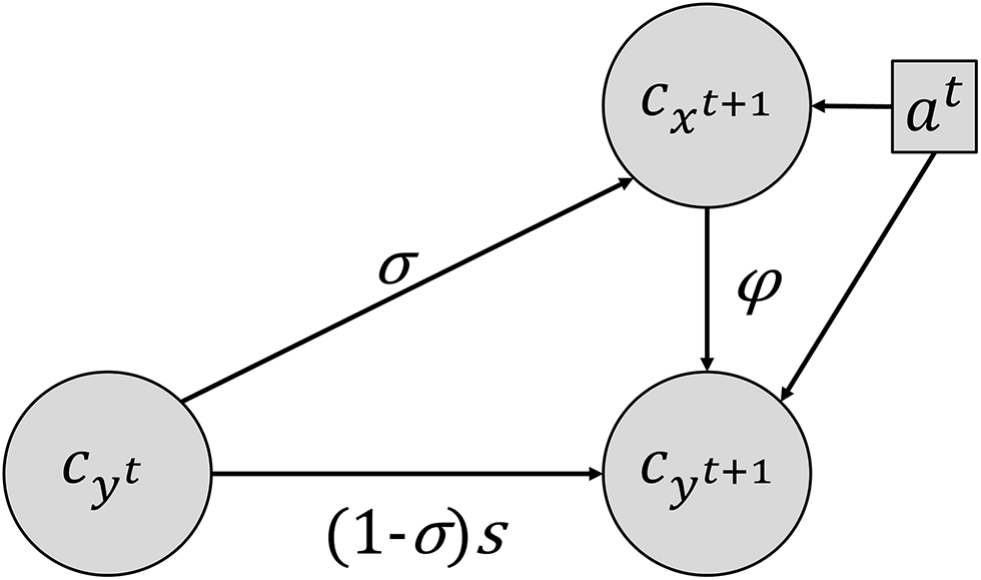
Hidden Markov Model for abundance classes. *c*_*x*_^*t+1*^, the abundance class of the emerged plants at time *t+1* depends on the abundance class of the weed population in the seed bank (*c*_*y*_^*t*^) and the management actions *a* ^*t*^ at time *t*. At time *t+1*, the abundance class of the weed population in the seed bank *c*_*y*_^*t+1*^ is the sum of the output of the interaction between the abundance class of the weed population in the seed bank (*c*_*y*_^*t*^) and management actions *a* ^*t*^ at time *t* and of the number of seeds produced by the emerged plants of this weed population at *t+1* (*c*_*x*_^*t+1*^). The three LHTs are the germination rate *σ*, the seed survival rate in the seed bank *s*, and the seed production number *φ*, i.e., the number of seeds from each emerged plant in the seed bank. The values of these three LHTs (*s*, *σ* and *φ*) depend on the management action *a* ^*t*^.

The HMM was used to model the dynamics of each weed species. *(c*_*x*_^*1*^, *c*_*x*_^*2*^, *…*, *c*_*x*_^*T*^ *)* are the observed variables corresponding to a time series of abundance classes of emerged weeds (*c*_*x*_^*t*^ belongs to *{1*,*2*,*3*,*4}*) and T is the time series length. The hidden variables correspond to the time series of abundance classes in the seed bank: *(c*_*y*_^*1*^, *c*_*y*_^*2*^, *…*, *c*_*y*_^*T*^ *)* with *c*_*y*_^*t*^ taking value in *{1*,*2*,*3*,*4*,*5*,*6}*. The temporal relationship between these variables is described by two conditional probabilities (Fig 1):

- $P_{a^{t}}(c_{x^{t+1}}|c_{y})$: the probability that at time *t+1*, there is an abundance class *c*_*x*_^*t+1*^ of emerged weeds when the abundance class in the seed bank at time *t* was *c*_*y*_^*t*^ and management actions, i.e., the crop species and its associated agricultural practices, *a* ^*t*^, were applied.
- $P_{a^{t}}(c_{y^{t+1}}|c_{x^{t+1}},c_{y})$: the probability that at time *t+1*, the abundance class in the seed bank is *c*_*y*_^*t+1*^ when the emerged weed abundance class *c*_*x*_^*t+1*^ is observed at time *t+1* and when, at time *t*, the abundance class in the seed bank was *c*_*y*_^*t*^ and management actions, *a* ^*t*^, were applied.

These two conditional probabilities depend on life history parameter triplets, (*σ*_*a*_, *s*_*a*_, *φ*_*a*_), which in turn are conditioned by the management actions *a*. More precisely, the conditional probability of emerged weeds, $P_{a^{t}}(c_{x^{t+1}}|c_{y})$, depends on the germination rate *σ*_a_ since only seeds that have germinated will produce a mature plant. *c*_*x*_^*t*^ does not contribute to *c*_*x*_^*t+1*^ since the studied species are annual. The conditional probability of the seed bank state transition, $P_{a^{t}}(c_{y^{t+1}}|c_{x^{t+1}},c_{y})$, depends on the germination rate *σ*_a_, the seed survival rate in the seed bank *s*_*a*_, and the seed production number *φ*_*a*_, i.e., the number of seeds from each emerged plant in the seed bank. The seed bank at a given time stage comprises the seeds that did not germinate and survived, plus the seeds produced by the mature plants. Therefore, in this HMM, the sets of triplets {(*σ*_*a*_, *s*_*a*_, *φ*_*a*_)}_*a⊰ A*_ for all possible management actions *(a⊰ A)* summarise the weed population dynamics in various crop conditions.

The two transition probabilities for emerged plant classes and the seed bank classes were derived by integrating simple equations over each class range that define the weed population dynamics based on count data (*X* ^*t*^, *Y* ^*t*^) of the emerged flora and the seed bank. To build the model, it was assumed that the number of emerged plants *X* ^*t+1*^ at time *t+1* followed a binomial distribution of parameters (*Y* ^*t*^, *σ*_*a*_), where *Y* ^*t*^ is the number of seeds in the seed bank at time *t*. It was also assumed that the number of dead seeds in the seed bank followed a binomial distribution with population size equal to the seed count *Y* ^*t*^, minus the number of germinated seeds (i.e., *X* ^*t+1*^) and probability (1—*s*_*a*_). Since intensive computer processing would be required to give an exact evaluation of the conditional probabilities, they were estimated by simulation. A complete description of the mathematical expression of the conditional probabilities and the method used for their estimation is given in S1 File.

### Maximum likelihood and Bayesian estimates of life history traits

The Maximum Likelihood estimates of the LHTs were evaluated as follows. For a given weed species in a given field, the probability of observing a time series *(c*_*x*_^*1*^, *c*_*x*_^*2*^, *…*, *c*_*x*_^*T*^ *)* of emerged weeds when the corresponding sequence of management actions is *(a* ^*0*^,*a* ^*1*^, *a* ^*2*^, *…*, *a* ^*T*^ *)*, is given by the formula:

$$L_{n}=\sum_{c_{y^{0}}\ldots c_{y^{T-1}}}P_{a^{0}}(c_{x^{1}}|c_{y^{0}})P\left(c_{y^{0}}\right)\prod_{t=2}^{T}P_{a^{t-2}}(c_{y^{t-1}}|c_{x^{t-1}},c_{y^{t-2}})P_{a^{t-1}}(c_{x^{t}}|c_{y^{t-1}})$$ (1)

where subscript *n* is the sample number. A sample is a time series and there are *N* samples for the *N* fields where the species was recorded. For a given species, it was assumed that the *N* time series were independent. The maximum likelihood estimators for the LHT are the values that maximise the log-likelihood:

$$logL=\sum_{n=1}^{N}\text{log}\left(L_{n}\right)$$ (2)

Therefore, since we considered four management actions, the output of this maximisation is a set of four triplets (*σ*_*a*_, *s*_*a*_, *φ*_*a*_) of LHT values, one for each management action *a* (equal to WC, OR, M or SF). In addition, to avoid introducing a *priori* assumptions on the initial distribution of abundance classes in the seed bank at *t =* 0, the *p(c*_*y*_^*0*^ *)* were considered as parameters, which were estimated by maximum log-likelihood, together with the LHT values. All calculations were carried out using the *R* package *FME* [20], based on the pseudo random search optimisation [21].

### Evaluation of the predictive efficiency by cross-validation

The quality of the LHT estimates obtained from the HMM model was evaluated by calculating the predictive efficiency, defined as the percentage of correct predictions of abundance classes of emerged plants. This was evaluated by cross-validation over four sub-datasets of 18 species and 82 fields selected at random (25% of the total number of fields). Each sub-dataset, in turn, represented the validation dataset, while the three others were merged and represented the training dataset. For a given training set, the maximum log-likelihood estimator was calculated using the above-mentioned *pseudoOptim* function with a large maximum number of iterations (50,000) and a relatively low precision criterion (1e-6) in the algorithm. The number of simulations used to evaluate transition probabilities (*K* in S2 File) was set to 30,000. The abundance class *c*_*x*_^*t*^ was then predicted as the mode of $P_{a^{t}}\left(c_{x^{t+1}}|c_{x^{t}}\right)$ calculated for the estimated LHT.

For each species, the model was tested to determine whether it correctly predicted the absence (class 1) and presence (class larger than 1) of species by calculating an average efficiency for predicting absence over the whole validation dataset (proportion of cases where class 1 was predicted when class 1 was observed) and an average predictive efficiency of presence (proportion of cases where a class above 1 was predicted when a class above 1 was observed). If the predictions differed from the observed classes, two average class errors were then estimated: one based on the prediction of absence (mean difference between the predicted class and “true” class 1) and the other on the prediction of presence (mean difference between the predicted class and the observed class for classes larger than 1).

### Relationships between LHT estimates and species characteristics

The quality of prediction of the model was also evaluated by investigating the relationships between estimated LHTs and current knowledge on weed ecology. Since the likelihood estimation procedure was highly time-consuming when applied to the whole data set, a faster estimation algorithm was used for the following analyses. Posterior distributions of the LHTs were estimated using a Bayesian approach with the Gibbs sampler algorithm. The Gibbs sampler requires a good starting point. Therefore, the maximum likelihood estimators obtained from the cross validation analysis were selected for initialisation. In practice, the posterior distribution parameters were obtained assuming uniform prior distributions and running the *gibbs* function of the *LearnBayes R* package with log-likelihood *logL* as the *posterior* density.

The relationship between the germination rate and the indicator species index (indicator value, IndVal) [22] of the 18 species was determined for each type of crop. IndVal is computed from a combination of a species' relative abundance with its relative frequency of occurrence within the various crop types based on botanical records. Consequently, within a given type of crop, we expected a positive correlation between this score and the estimated germination rate since the most frequent species would also be those that easily germinate in these conditions. IndVal indices were computed (using the *indicspecies* R package, [22]) to discriminate autumn/winter and spring/summer weeds by measuring species specificity and frequency in several crop types. The relevance of the model predictions was also tested by analysing the correlation between LHT estimates (averaged over the four crop types) and two functional traits: the seed mass, which is related to reproduction [23], and the seed coat thickness, which is related to seed persistence in the seed bank [24]. Depending on total allocation trade-offs [20], the average seed production per plant (i.e., number of seeds) was expected to be negatively correlated with the per capita seed mass. The average seed survival rate was expected to be positively correlated with the seed coat thickness as shown by Gardarin et al. [24]. Weed functional trait values were extracted from the WEED-DATA database (INRA, S. Gaba pers. comm.).

### LHT combination and growth rate

LHT estimates were used to identify the triplets (*σ*_*a*_, *s*_*a*_, *φ*_*a*_) which represent the life history strategy that gives positive growth rates for the management conditions, *a*. Species growth rates were estimated from the three LHTs using a Leslie matrix framework that incorporates the LHTs of the weed species into a structured population model [25]:

$${\left[\begin{array}{c} n_{a_{seed}}\\ n_{a_{plant}} \end{array}\right]}_{t+1}\;=\;\left[\begin{array}{cc} s_{a}\left(1-\sigma_{a}\right)+\sigma_{a}\varphi_{a} & 0\\ \sigma_{a} & 0 \end{array}\right]{\left[\begin{array}{c} n_{a_{seed}}\\ n_{a_{plant}} \end{array}\right]}_{t}$$

The asymptotic growth rate *λ*_*a*_ (i.e., when the equilibrium state is reached) is the dominant eigenvalue of the Leslie matrix for each management action *aϵA*. A species has a positive growth rate when *λ*_*a*_ is greater than 1; otherwise it is negative (*λ*_*a*_ <1) or stable (*λ*_*a*_ = 1). Since weed populations are disturbed as a result of, for example, herbicide applications or soil surface preparation, we also explored the deviation between the long-term and the transient population growth rates by computing the damping ratio (see S3 File for more details), which measures the speed of convergence to the asymptotic growth rate. Finally, the relative contribution of LHT (*σ*_*a*_, *s*_*a*_, *φ*_*a*_) on species growth rates was quantified by a random forest analysis performed for each species and type of crop (*randomForest* R package).

## Results

### Model predictive efficiency assessment by cross-validation

For each species, the predictive efficiency of the model varied between 93% and 100% (mean = 96.83%) for predicting the absence of species. However, the model tended to overestimate the abundance class 1, i.e., the absence of weed species. As a direct consequence, better estimates were obtained for situations with no weed species than for situations with few or many weeds (Table 1). Consequently, the prediction efficiency for the presence of species varied between 0% and 67% (mean = 19.72%) (Table 1) and was slightly correlated with the species occurrence in the botanical records (*ρ* = 0.42, *P*-value = 0.083).

**Table 1:** Predictive efficiency and class attribution error.

EPPO Code	Latin Name	Field occurrence (n = 329)	Record occurrence (n = 1191)	Average predictive efficiency for absence	Average predictive efficiency for presence	Average class error for absence	Average class error for abundance
**ALOMY**	*Alopecurus myosuroides Huds*.	39.8%	20.8%	0.96	0.27	1.48 (0.10)	-1.24 (0.06)
**ANGAR**	*Anagallis arvensis* L.	43.1%	15.4%	1	0	n.a.^a^	-1.17 (0.03)
**CHEAL**	*Chenopodium album* L.	74.1%	46.0%	0.93	0.67	1.67 (0.08)	-0.58 (1.07)
**FUMOF**	*Fumaria officinalis* L.	32.2%	12.7%	1	0	n.a.^a^	-1.14 (0.04)
**GALAP**	*Galium aparine* L.	59.5%	30.8%	0.96	0.17	1.08 (0.05)	-1.20 (0.03)
**MERAN**	*Mercurialis annua* L.	41.6%	23.8%	0.97	0.37	1.23 (0.09)	-1.06 (0.07)
**PAPRH**	*Papaver rhoeas* L	45.5%	20.9%	0.98	0.12	1 (0)	-1.16 (0.03)
**POAAN**	*Poa annua* L	38.9%	19.9%	0.98	0.24	1.62 (0.125)	-1.31 (0.07)
**POLAV**	*Polygonum aviculare* L	54.7%	27.2%	0.99	0.04	1 (0)	-1.34 (0.04)
**POLCO**	*Fallopia convolvulus* L	44.9%	20.8%	0.96	0.17	1.10 (0.06)	-1.19 (0.06)
**SENVU**	*Senecio vulgaris* L	66.5%	35.0%	0.95	0.10	1.12 (0.07)	-1.15 (0.02)
**SINAR**	*Sinapis arvensis* L.	37.3%	19.8%	0.97	0.27	1.12 (0.08)	-1.13 (0.05)
**SOLNI**	*Solanum nigrum* L.	56.5%	26.7%	0.93	0.53	1 (0)	-1.37 (0.05)
**SONAS**	*Sonchus asper* L.	50.7%	22.0%	0.99	0.03	1 (0)	-1.08 (0.03)
**SONOL**	*Sonchus oleraceus* L.	27.0%	10.6%	1	0	n.a.^a^	-1.08 (0.03)
**STEME**	*Stellaria media* L.	48.6%	25.6%	0.96	0.18	1.29 (0.09)	-1.42 (0.05)
**VERHE**	*Veronica hederifolia* L.	52.2%	25.2%	0.94	0.28	1.02 (0.03)	-1.29 (0.04)
**VERPE**	*Veronica persica* L.	51.6%	24.1%	0.96	0.11	1.21 (0.08)	-1.24 (0.04)
**Mean**		**48%**	**23.7%**	**0.97**	**0.19**	**1.19 (0.057)**	**-1.17 (0.1)**

This table gives the average predictive efficiencies for the absence and presence of species, the average class error for absence class (class 1) and for abundance classes (class 2 to 4) when using estimated LHT parameters and estimated distribution of the seed bank state at *t* = 0. Field occurrence and record occurrence are the percentages of fields and of botanical records in which the species was recorded, respectively. Values in parentheses give the standard average class error.

^a^ Data were not available.

Differences between the predicted and the observed abundance classes were typically of one abundance class (1.18 abundance class on average, Table 1). When the species was absent and incorrectly predicted, the model tended to predict an abundance class 2 (1 to 2 ind/m²) and, conversely, when the species was present and incorrectly predicted, its abundance class was generally underestimated by one abundance class (Table 1).

Detailed parameter distribution estimates (probability distribution of seed bank states at *t* = 0 and LHT) obtained over the whole dataset by the Gibbs sampler for the 18 weed species are presented in the Supplementary Material (S3 and S4 Tables, S2 and S3 Figs).

### Relationships between life history traits and indicator values and seed functional traits

**Fig 2:**
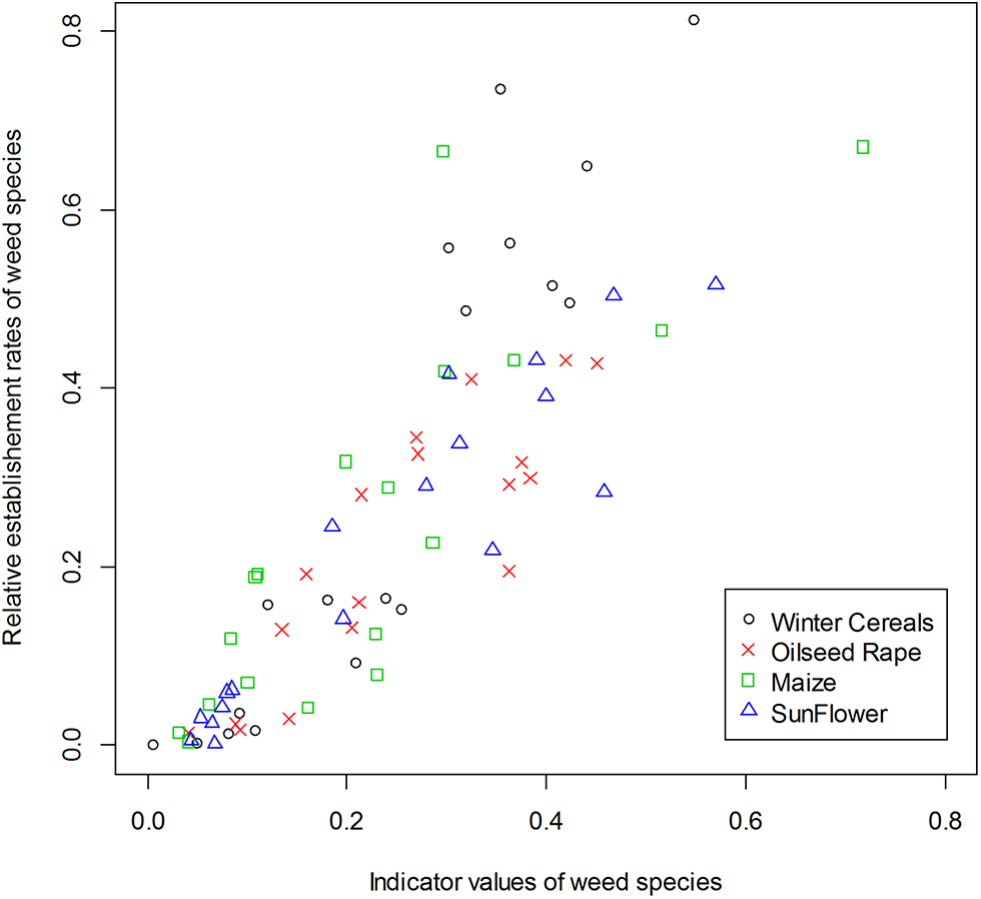
Relationship between relative germination rates and IndVal indicator values for each species with different crop types. Symbols indicate the crop type. Positive correlations were highly significant. Winter cereals = circle (Spearman’s correlation unilateral test, *ρ* = 0.93, *P*-value < 2.2e-16), Oilseed rape = cross (*ρ* = 0.88, *P*-value < 2.2e-16), Maize = square (*ρ* = 0.88, *P*-value < 2.2e-16) and Sunflower = triangle (*ρ* = 0.91, *P*-value < 2.2e-16).

**Fig 3:**
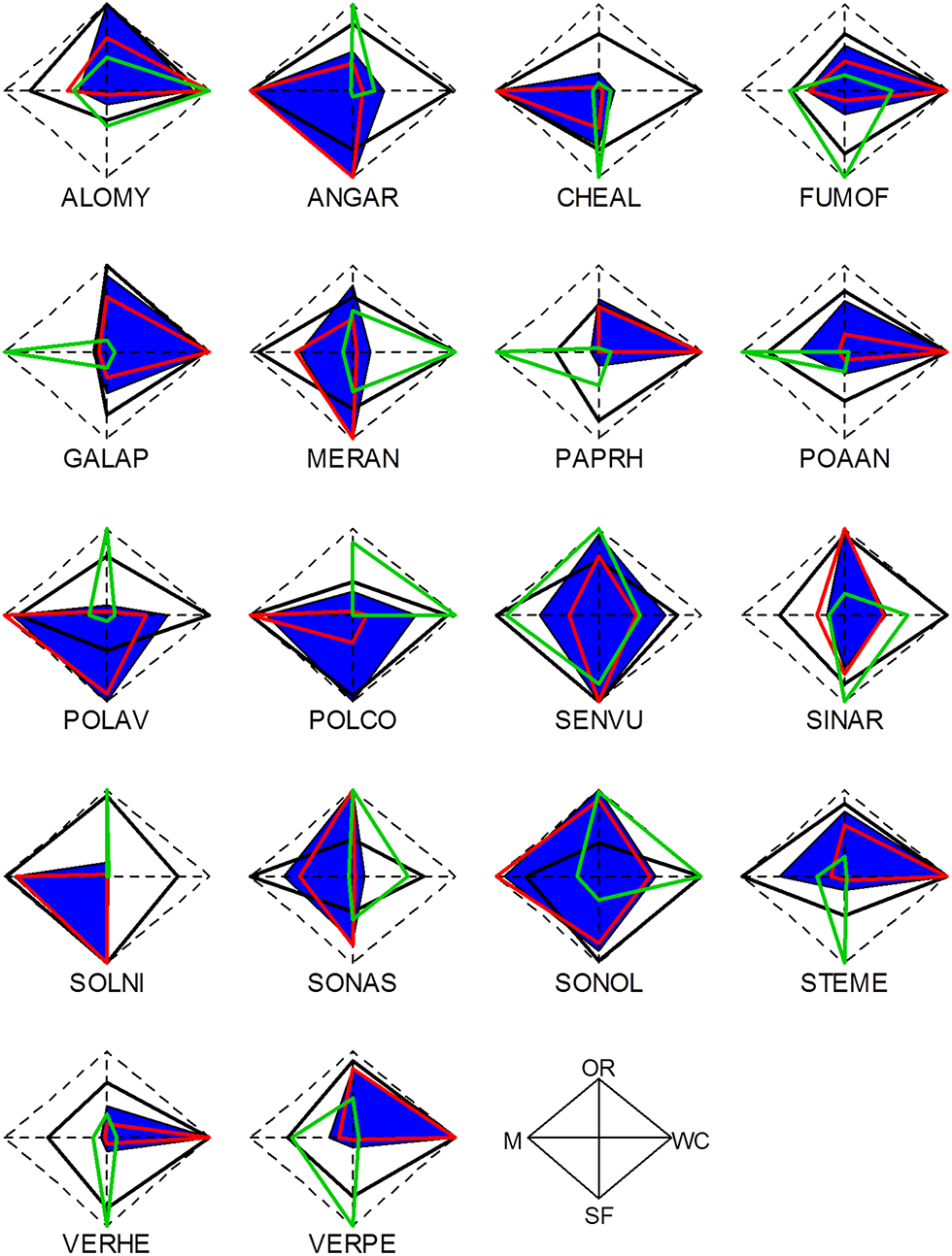
Life history traits and IndVal of weed species with the four crop types. Species survival rates (*s*), germination rates (*σ*) and seed production per plant (*φ*) are presented by black, red and green polygons for four crop types, respectively (WC = winter cereals, OR = oilseed rape, M = maize and SF = sunflower). Values are scaled by dividing each value by the maximum value with the four crop types. For each species, each scaled Life History Trait (LHT) varies between 0 and 1 and the polygon is shifted in the direction of the crop(s) where it has its highest estimated success. Dashed polygons represent values equal to 1 for the three life history traits, i.e., maximum value for all species for each LHT. Blue polygons represent the indicator values (IndVal) of species with the four crop types (scaled by the maximum value of species with the four crop types).

We observed the expected relationships between the estimated values of LHT and both the indicator values (IndVal) and the functional traits. For all crop types, the relative establishment rate of the species was highly positively correlated with the species IndVal (Figs 2 and 3). The estimated average seed production (*φ*_*a*_) was not correlated with seed mass (Spearman’s correlation unilateral test, n = 18, *ρ* = -0.31, *P*-value = 0.104). Conversely, the estimated average seed survival rate (*s*_*a*_) was positively correlated with the seed coat thickness (Spearman’s correlation unilateral test, n = 9, *ρ* = 0.73, *P*-value = 0.015) even though the sample size was small (seed coat thickness values were only available for half of the species; S4 Fig).

### Life History Strategies

**Fig 4:**
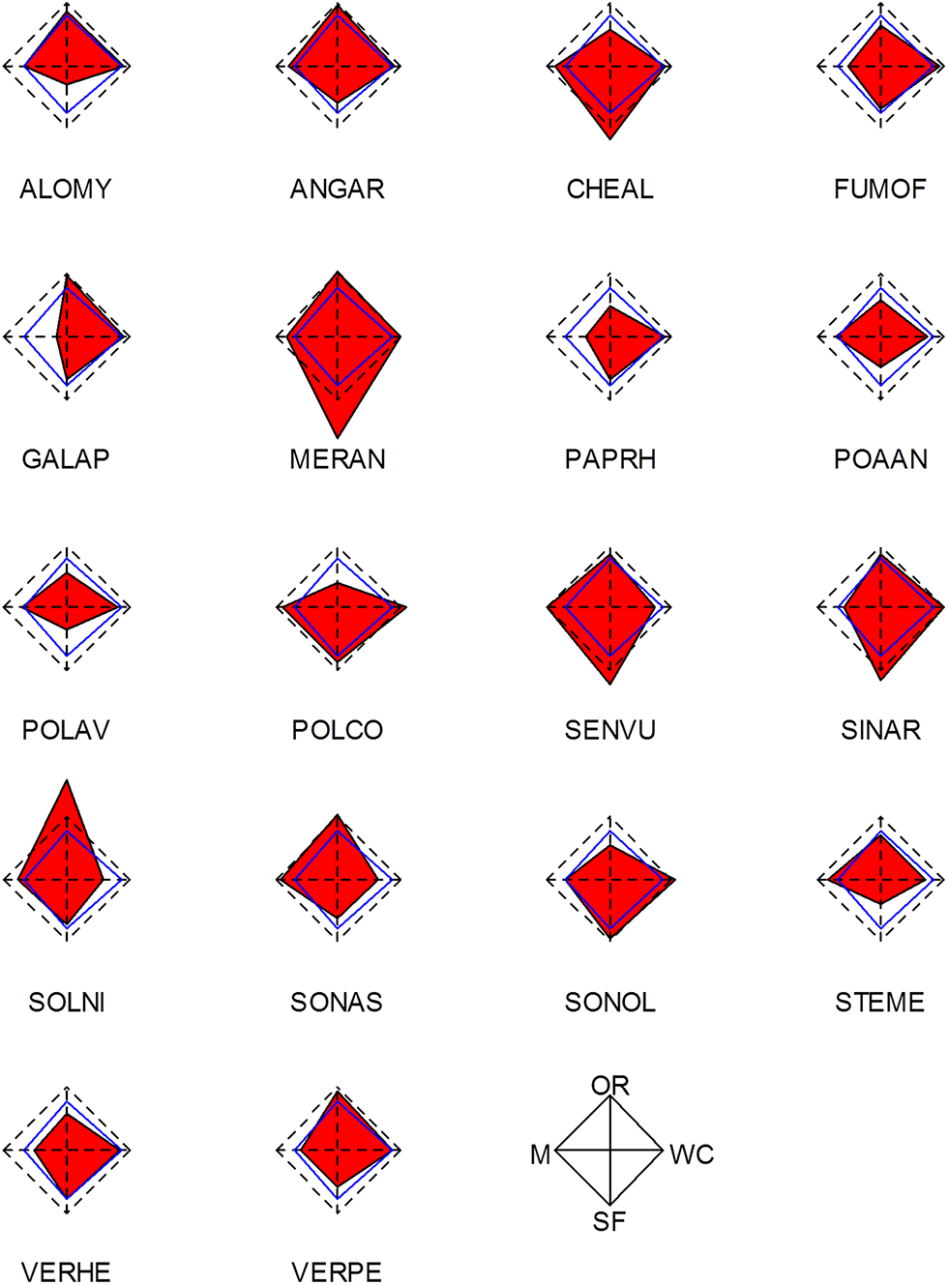
Growth rates of seed banks with the four major crop types. Red polygons represent the growth rates of the weed species with the four crop types (WC = winter cereals, OR = oilseed rape, M = maize and SF = sunflower), as indicated by the bottom right polygon. For each species, the dashed polygon represents a growth rate equal to 1 and the centre corresponds to a growth rate equal to zero. Blue polygons represent the mean growth rates of the 18 species in each crop type. Species names are indicated by EPPO codes.

There were high interspecific and intraspecific variations between LHT estimates and growth rates and none of the 18 species had similar LHT values in the four crop types (see Figs 3 and 4, built from S2 and S3 Figs). Of the 18 species, only *Mercurialis annua* L. had a positive asymptotic growth rate in all four crop types. Eight species had a positive growth rate in at least one crop type, while ten species had a negative growth rate in the four crop types. The highest number of species with a positive growth rate included winter cereals and sunflower (4). Conversely, around 90% of the species (17) showed negative growth rates with maize (Fig 4). The damping ratio (S5 Table) revealed that most of the species had a low convergence speed to the asymptotic growth rate, suggesting that these weed populations are not yet in the equilibrium state and exhibit long transient dynamics. This was expected due to the high disturbance regimes in arable fields. However, few species populations, mostly in winter cereals, seemed closer to the equilibrium (e.g., damping ratio of 256 for *Solanum nigrum* L. in winter cereals; S5 Table).

Within a crop type, different combinations of LHTs showed positive growth for various species. For example, *M*. *annua* and *Senecio vulgaris* L. showed positive growth rates with sunflower but with different combinations of LHTs (Fig 3). A comparative analysis of species growth rates for crop types (Fig 4) and the mean species LHTs for crop types (Fig 3) showed that different combinations of LHTs could lead to similar growth rates, revealing variations in life history strategies between crop types for a given species and between species for a given crop type. Growth rates were positively correlated with germination rates in maize (*ρ* = 0.92, *P*-value < 2.2e-16) and with seed survival rates in winter cereals (Spearman’s correlation bilateral test, *ρ* = 0.69, *P*-value = 1.96e-3). Furthermore, random forest and regression tree analysis revealed that the survival rate (*s*_*a*_) was the most important LHT for determining the species growth rate for a given crop type.

**Fig 5:**
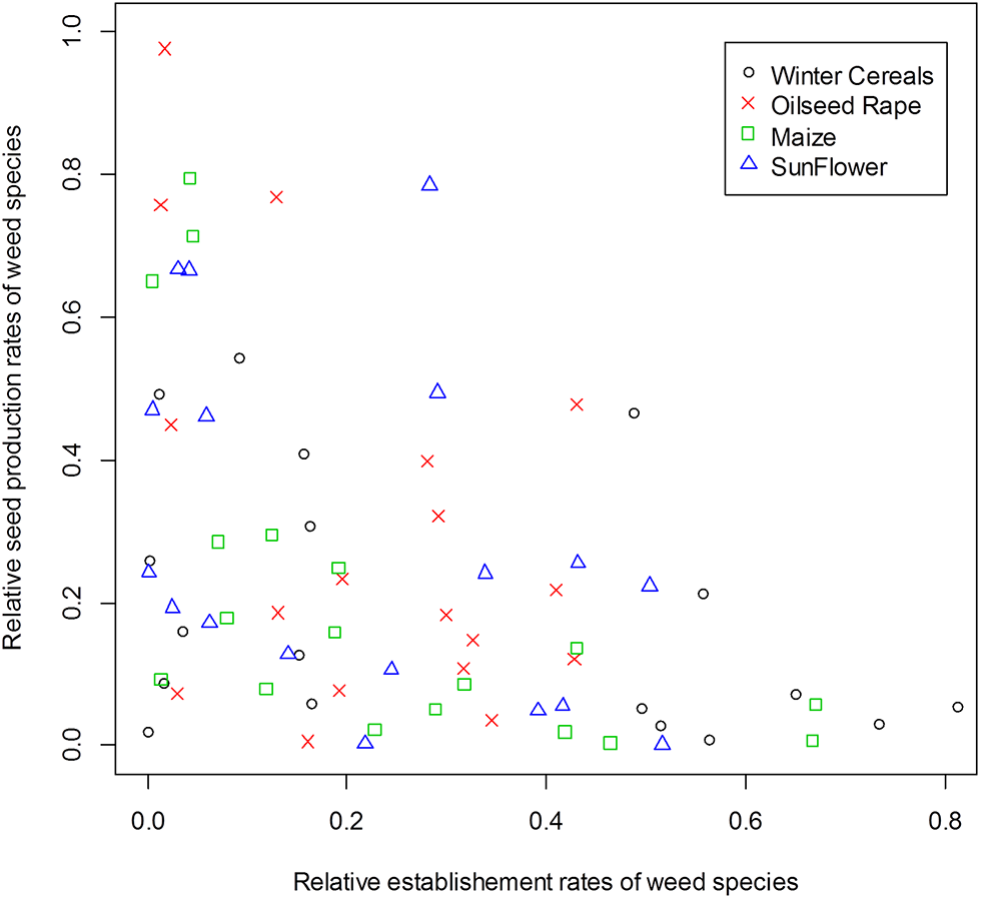
Relationship between the relative germination rate and relative seed production per plant in each crop type. Symbols indicate the crop type (winter cereals = circle, oilseed rape = cross, maize = square, sunflower = triangle). Relative germination rates were not correlated with the relative seed production rates in winter cereals (Spearman correlation test, *ρ* = -0.39, *P*-value = 0.103), oilseed rape (*ρ* = -0.32, *P*-value = 0.185), maize (*ρ* = -0.76, *P*-value = 3.34e-4) and sunflower (*ρ* = -0.40, *P*-value = 0.094), but were negatively correlated in maize.

All LHT values for a given species varied between crop types. The highest variations were observed for species seed production (*φ*_*a*_) and germination rates (*σ*_a_), which varied significantly between crop types, ranging from values close to 0 to a maximum seed production or germination rate depending on the crop type (Fig 3). Relative species germination rates were not correlated with relative seed production rates in winter cereals (Spearman correlation test, *ρ* = -0.39, *P*-value = 0.103), oilseed rape (*ρ* = -0.32, *P*-value = 0.185), and sunflower (*ρ* = -0.40, *P*-value = 0.094), but were negatively correlated in maize (*ρ* = -0.76, *P*-value = 3.34e-4) (Fig 5). Overall, no species had maximum values of all LHTs in all four crop types (Fig 3).

## Discussion

This study showed that even in the absence of observation of the seed bank, it is possible to build a model that makes it possible to characterise and distinguish weed dynamics under different crop and weed management practices. The model is a hidden Markov model where the influence of the hidden stage, the seed bank, is described by three Life History Trait (LHT) parameters. This simple model is not dedicated to prediction (extension would be required as discussed below) but to the study of the qualitative variations of the LHT estimates under different management practices.

The time series dataset that was used to estimate the three LHTs of the HMM- seed survival, plant establishment and seed production—and for identification of weed life history strategies in the four crop types is a contribution to weed science and ecology. The data enables researchers to provide qualitative information about life history traits and strategies and how they vary between crops and species. It is particularly helpful when empirical data is missing or derived from standardised experimental conditions that may differ from management conditions and include only limited contrasting environmental conditions.

Correlations were found between the estimated LHTs and functional traits and between the estimated LHTs and ecological characteristics. Consistent with Gardarin et al. [24], we found a significant correlation between seed coat thickness and seed survival in the seed bank. This conclusion was in accordance with an expected trade-off between seed size and number [26]. Moreover, a significant negative correlation between estimated seed production and germination rate was observed. Such a pattern may reveal two strategies. First, species with higher seed production have small seeds, which generally have high seedling mortality due to a lower competition ability. Second, species with a lower seed production but with heavier seeds may have a better chance to germinate and/or establish themselves. However, part of this trade-off may come from the indirect effect of management practices. Farmers generally adapt their weed control strategies to deal with the most abundant, pernicious weed species. Weed species with low abundance may not be targeted by mechanical or chemical treatment, which results in them escaping with an increased capacity to complete their life cycle by producing seeds. Their low density and good access to nutrients are also advantageous.

Another result was that indicator species for a crop type, i.e., those with high indicator values (IndVal), generally had higher germination rates. This result is consistent with previous studies (e.g., Gunton et al. 2011 [14]) that showed the importance of synchrony between weed and crop germinations. Within the ephemeral environment of an arable field with a short window for growth, successful weeds normally germinate around the time the crop is sown and complete their reproductive efforts before the crop is harvested. The approach based on an explicit model of the seed bank dynamics is complementary to the IndVal values, which are calculated for emerged plants without any reference to seed bank dynamics, and gives a better picture of the role of crop sequences on weed abundance.

Another interesting result obtained from the LHT estimates is that different combinations of LHT values can provide a positive growth rate. This pattern was observed across species, suggesting that more than one strategy can be beneficial within the weed community in response to particular agricultural practices as well as within species with different strategies that depend on the crop type. This pattern might be explained by intraspecific weed variation, which has been previously shown within and between fields [27]. LHT estimates could be further used to simulate and test weed population dynamics according to specific crop sequences by computing the population asymptotic growth rate and the damping ratio. The HMM therefore has an interesting explanatory power for studying the above-ground population dynamics of weeds in agroecosystems, especially since it accounts for the contribution of the hidden seed bank.

The HMM developed relies on several assumptions. The first one is that seeds in the bank are indistinguishable, with the consequence that all seeds have the same probability to survive from one year to the next, regardless of when they enter the seed bank. The consequence is a potential overestimation of a weed’s lifetime. However, removing this assumption would be complex: it would lead to a non-Markovian model that would be much more complex to estimate. The following two assumptions, which are easily broadened, can explain why the quality of prediction of the presence of weeds remains low. First, by construction, the conditional distribution of abundance classes of emerged weeds was unimodal. However, observations of absence of a species (class 1) were over-represented in the dataset and classes 1 and 2 were probably too close to be distinguished (especially from heterogeneous fields). When training the model, this leads to overfitting the abundance class 1 while other classes are underestimated. This problem could be tackled using zero-inflated distributions instead of Poisson and Binomial distributions for counts in the underlying HMM. The second strong hypothesis of the model is that is does not account for weed dispersal from neighbouring fields or semi-natural habitats. However, several studies have shown that weed dispersal occurs frequently beyond the crop field scale [15, 28]. Ignoring spatial weed dispersal in the HMM is probably (only partly) compensated for by overestimating the seed bank. Biases in parameter estimation (colonisation and extinction rate) were recently observed when not taking account of the seed bank in a recent study using a HMM to explore plant population dynamics [17]. Therefore, if spatial dispersion is not taken into account it may lead to a similar bias. A spatial version of the HMM would make it possible to compare the relative importance of spatial dispersal and the seed bank (temporal dispersal) on weed dynamics. Spatial dispersal could be modelled using Dynamic Bayesian Networks [29] that describe a network of interacting crop fields, as in Peyrard et al. (2007) [30] or Tixier et al. (2013) [31].

## Conclusions

This study proposes a method to account for the influence of the hidden seed bank on weed dynamics in the absence of seed bank data, using HMM. This approach can be useful in increasing the knowledge about species ecology. It can also provide an easy access to LHT estimates, which is of high biological value for capturing the dynamics of weeds but could also be applied to many plant and animal species with an unobservable life form that have remained poorly understood because of the lack of suitable methodological approaches.

The HMM approach could also be used to design sustainable management policies. Indeed, an interesting feature of the HMM is that the simulation is easy, making it possible to extensively explore possible management scenarios, as has already been successfully done in the ecological conservation literature [32]. Social, environmental and economic consequences of each scenario could then be compared. To do so, the HMM model should be extended with a quantification of the impact of weeds and weed dynamics on crop production and biodiversity, and a model of the value the different stakeholders attribute to the different configurations of the triplet, “crops × weeds × practices”. Beyond the design by simulation of a limited set of scenarios, we could also use the HMM to design management strategies by optimization, which would make it possible to search a larger space of possible strategies. This could be done by embedding the model into a framework for sequential decision under uncertainty. The resulting extension of the HMM would be a Partially Observed Markov Decision Process (POMDP) ([33], [34]). This framework has already been successfully applied in the ecological conservation literature [32] and would be relevant for testing or optimizing various weed management strategies, while taking the influence of the seed bank into account.

## Supporting Information

S1 Fig Locations of the fields in which weeds were surveyed by the French Biovigilance Network. (TIF)

S2 Fig Distribution of parameters obtained with the Gibbs sampler algorithm for the 18 studied species in the four crop types. Seed bank distribution. Yk stands for the probability that the seed bank is in class k at *t* = 0 (WC = winter cereals, OR = oilseed rape, M = maize and SF = sunflower).
(TIF)

S3 Fig Distribution of parameters obtained with the Gibbs sampler algorithm for the 18 studied species in the four crop types. Life history traits (WC = winter cereals, OR = oilseed rape, M = maize and SF = sunflower).
(TIF)

S4 Fig Relationship between average LHT values of species and functional traits. The average seed production tends to be negatively correlated to the average seed mass of the species (Spearman’s correlation unilateral test, n = 18, *ρ* = -0.31, *P*-value = 0.104), and the average seed survival rate of a species is positively correlated to the average seed coat thickness of the species (Spearman’s correlation unilateral test, n = 9, *ρ* = 0.73, *P*-value = 0.015).
(TIF)

S1 Table Percentage of fields used for the estimation of life history trait values. Values correspond to the proportion of fields where the crop type (WC = winter cereals, OR = oilseed rape, M = maize and SF = sunflower) has been sown at least once and the species has been observed at least once in the crop sequence. The species in bold print were those that were retained for our study.
(PDF)

S2 Table Current knowledge about weed species. Ind_WC, Ind_OR, IND_M and Ind_SF represent the indicator values (Indval) of species in winter cereals (WC), oilseed rape (OR), maize (M) and sunflower (SF), respectively.
(PDF)

S3 Table Estimated distribution of seed bank states at *t* = 0. Yk stands for the probability that the seed bank is in class k at *t* = 0. Means and standard deviations are reported. These values were obtained using the Gibbs sampler algorithm.
(PDF)

S4 Table Estimated Life History Traits. Means and standard deviations of life history trait distributions obtained by the Gibbs sampler algorithm.
(PDF)

S5 Table Damping Ratio associated with the Leslie matrix model for each weed species within a given crop type. WC = winter cereals, OR = oilseed rape, M = maize and SF = sunflower.
(PDF)

S1 File HMM model on counts. (PDF)

S2 File HMM model on abundance classes. (PDF)

S3 File Population dynamics. (PDF)

S4 File Coordinates of the fields in which weeds were surveyed by the French Biovigilance Network. Coordinates are given in the Lambert II reference system (epsg:27572).
(CSV)
