# Supplementary figures and images for: Dynamics of Weeds in the Soil Seed Bank: A Hidden Markov Model to Estimate Life History Traits from Standing Plant Time Series
Source: PLoS One. 2015 Oct 1;10(10):e0139278. doi: 10.1371/journal.pone.0139278 (PMC4591344; doi:10.1371/journal.pone.0139278)

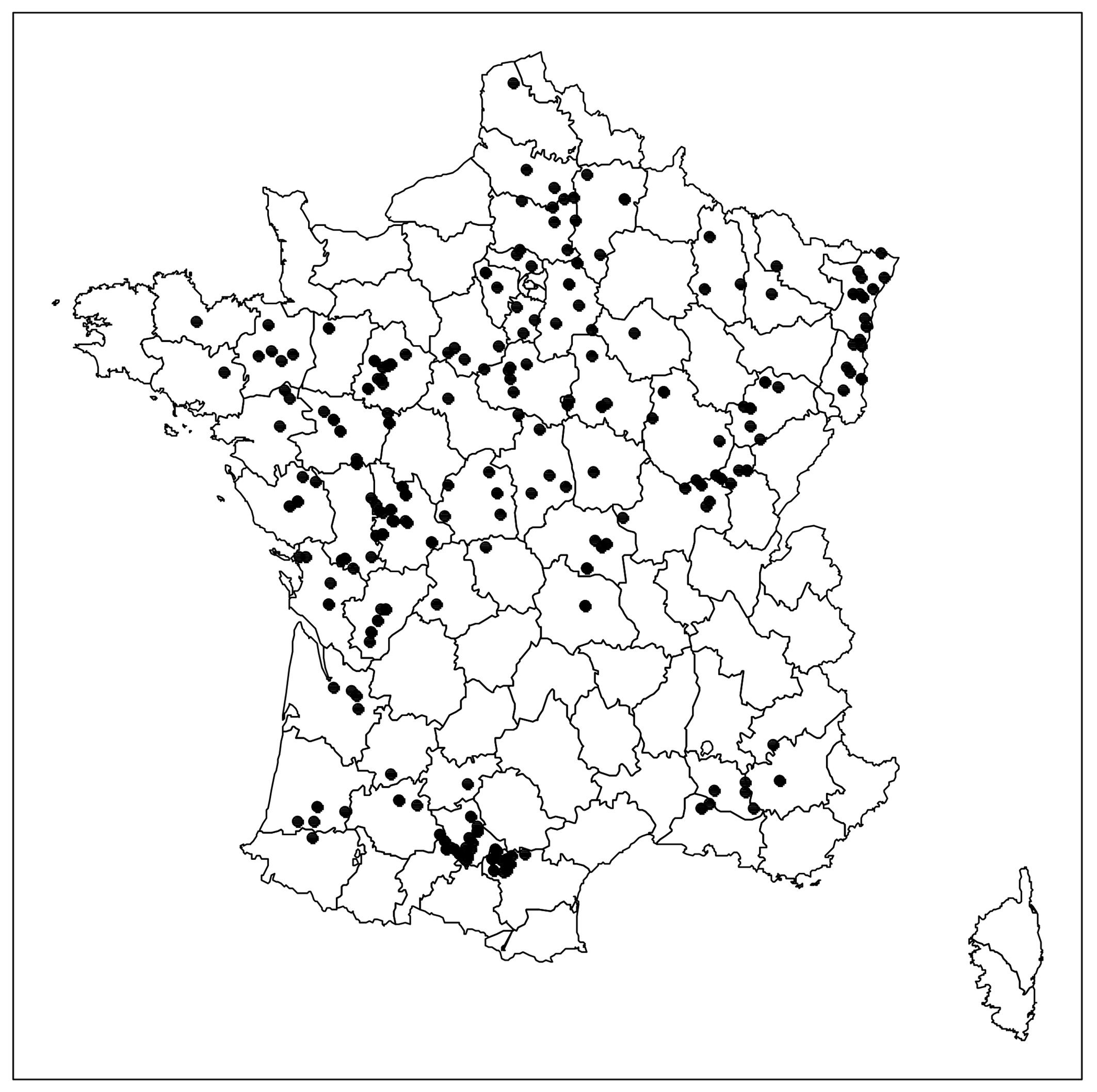

Supplement: S1 Fig — (TIF) [file pone.0139278.s001.tif]

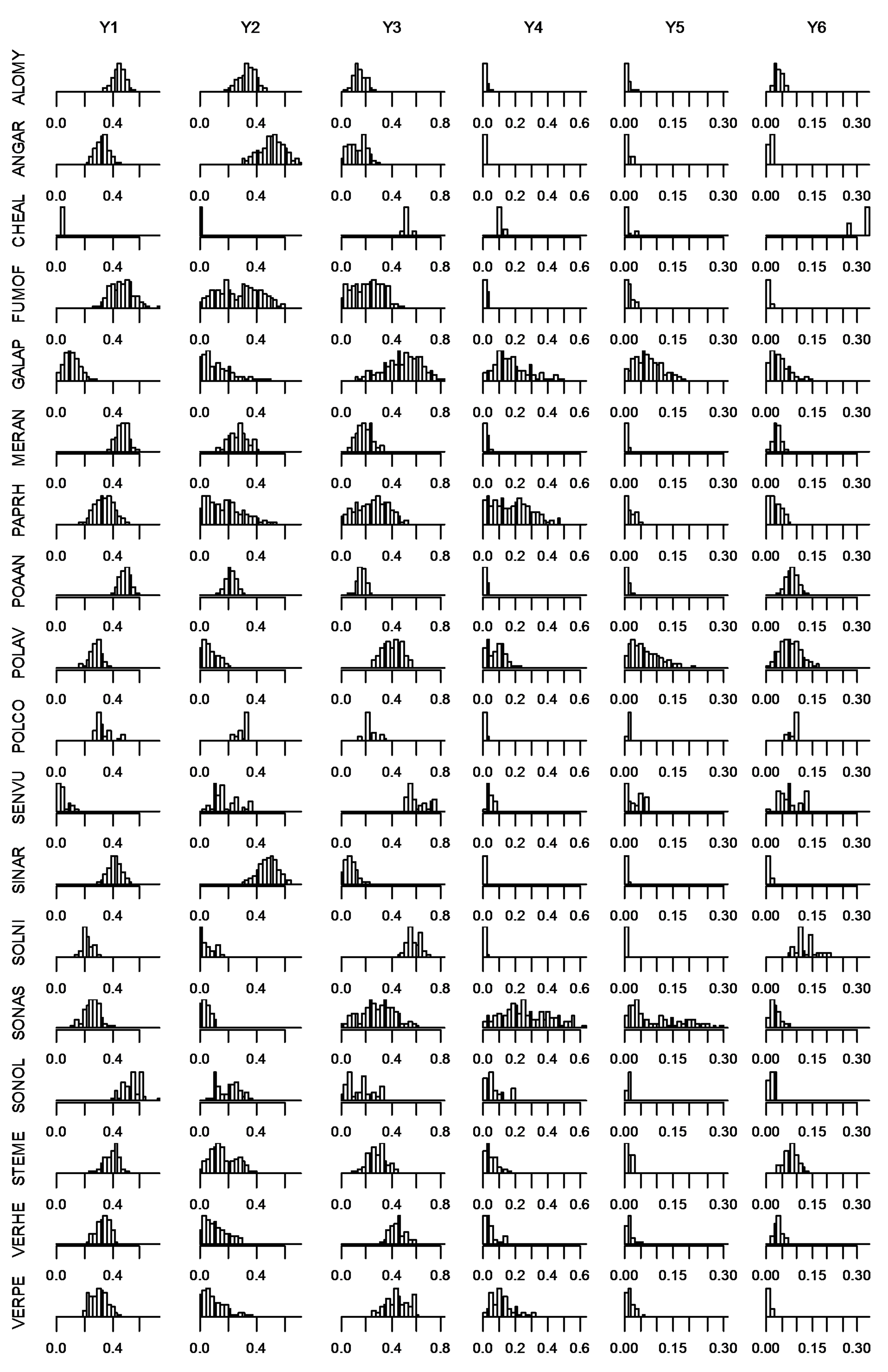

Supplement: S2 Fig — Seed bank distribution. Yk stands for the probability that the seed bank is in class k at t = 0 (WC = winter cereals, OR = oilseed rape, M = maize and SF = sunflower). (TIF) [file pone.0139278.s002.tif]

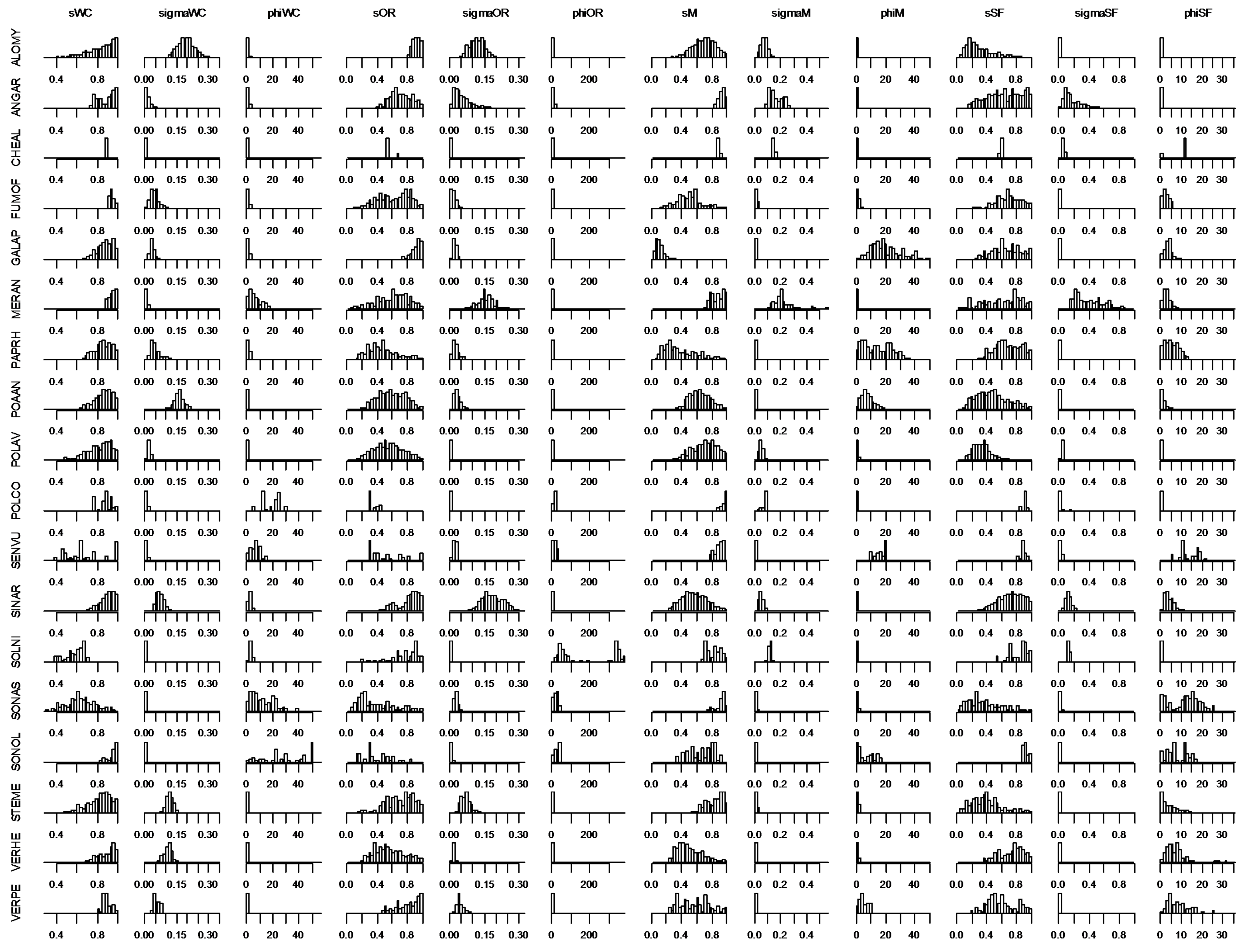

Supplement: S3 Fig — Life history traits (WC = winter cereals, OR = oilseed rape, M = maize and SF = sunflower). (TIF) [file pone.0139278.s003.tif]

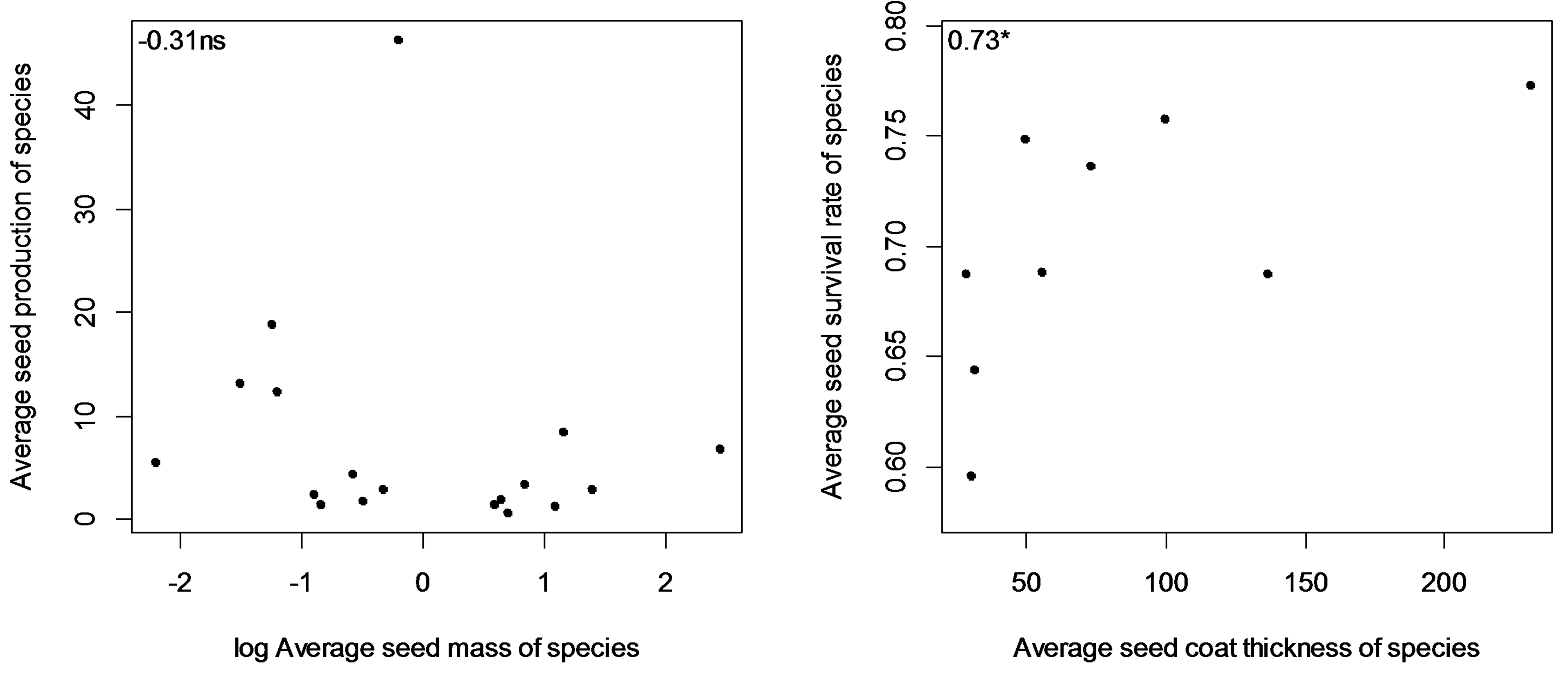

Supplement: S4 Fig — The average seed production tends to be negatively correlated to the average seed mass of the species (Spearman’s correlation unilateral test, n = 18, ρ = -0.31, P-value = 0.104), and the average seed survival rate of a species is positively correlated to the average seed coat thickness of the species (Spearman’s correlation unilateral test, n = 9, ρ = 0.73, P-value = 0.015). (TIF) [file pone.0139278.s004.tif]
