# Supplementary material for: Dynamics of Weeds in the Soil Seed Bank: A Hidden Markov Model to Estimate Life History Traits from Standing Plant Time Series
Source: PLoS One. 2015 Oct 1;10(10):e0139278. doi: 10.1371/journal.pone.0139278 (PMC4591344; doi:10.1371/journal.pone.0139278)
Supplement: S1 Table — Values correspond to the proportion of fields where the crop type (WC = winter cereals, OR = oilseed rape, M = maize and SF = sunflower) has been sown at least once and the species has been observed at least once in the crop sequence. The species in bold print were those that were retained for our study. (PDF) [file pone.0139278.s005.pdf]

EPPO Code	WC	OR	M	SF
**ALOMY**	**0.39**	**0.19**	**0.12**	**0.10**
AMARE	0.31	0.05	0.25	0.17
**ANGAR**	**0.36**	**0.10**	**0.23**	**0.15**
APHAR	0.20	0.12	0.06	0.05
ATXPA	0.22	0.07	0.13	0.10
CAPBP	0.33	0.13	0.21	0.09
**CHEAL**	**0.62**	**0.16**	**0.41**	**0.27**
DIGSA	0.10	0.00	0.14	0.04
ECHCG	0.29	0.05	0.28	0.14
EPHHE	0.24	0.11	0.09	0.12
**FUMOF**	**0.30**	**0.11**	**0.11**	**0.11**
**GALAP**	**0.58**	**0.25**	**0.16**	**0.21**
GERDI	0.23	0.13	0.08	0.07
LACSE	0.15	0.05	0.03	0.08
LAMPU	0.18	0.08	0.09	0.06
MATCH	0.28	0.13	0.11	0.07
**MERAN**	**0.37**	**0.13**	**0.19**	**0.18**
**PAPRH**	**0.44**	**0.15**	**0.13**	**0.16**
**POAAN**	**0.34**	**0.10**	**0.20**	**0.10**
**POLAV**	**0.48**	**0.13**	**0.28**	**0.20**
**POLCO**	**0.40**	**0.13**	**0.20**	**0.19**
POLPE	0.34	0.09	0.25	0.16
RAPRA	0.24	0.11	0.08	0.10
**SENVU**	**0.56**	**0.20**	**0.32**	**0.24**
**SINAR**	**0.35**	**0.19**	**0.12**	**0.15**
**SOLNI**	**0.45**	**0.12**	**0.34**	**0.23**
**SONAS**	**0.41**	**0.16**	**0.28**	**0.16**
**SONOL**	**0.21**	**0.10**	**0.14**	**0.10**
**STEME**	**0.41**	**0.16**	**0.26**	**0.10**
TAROF	0.16	0.06	0.11	0.07
**VERHE**	**0.51**	**0.20**	**0.16**	**0.19**
**VERPE**	**0.49**	**0.18**	**0.16**	**0.17**
