# Supplementary material for: Dynamics of Weeds in the Soil Seed Bank: A Hidden Markov Model to Estimate Life History Traits from Standing Plant Time Series
Source: PLoS One. 2015 Oct 1;10(10):e0139278. doi: 10.1371/journal.pone.0139278 (PMC4591344; doi:10.1371/journal.pone.0139278)
Supplement: S2 Table — Ind_WC, Ind_OR, IND_M and Ind_SF represent the indicator values (Indval) of species in winter cereals (WC), oilseed rape (OR), maize (M) and sunflower (SF), respectively. (PDF) [file pone.0139278.s006.pdf]

EPPO Code	Ind_WC	Ind_OR	Ind_M	Ind_SF
ALOMY	0.3192	0.3839	0.1079	0.0648
ANGAR	0.0922	0.1347	0.2985	0.3025
CHEAL	0.1074	0.1425	0.7172	0.4575
FUMOF	0.3017	0.1596	0.1100	0.0798
GALAP	0.4227	0.3754	0.0617	0.1963
MERAN	0.0810	0.3632	0.2415	0.4672
PAPRH	0.4406	0.2693	0.0409	0.0676
POAAN	0.3539	0.2122	0.1603	0.0849
POLAV	0.2383	0.0404	0.3679	0.3996
POLCO	0.2088	0.0880	0.2959	0.3462
SENVU	0.2554	0.3621	0.2291	0.3904
SINAR	0.1804	0.4505	0.0830	0.2791
SOLNI	0.0059	0.0924	0.5151	0.5705
SONAS	0.0489	0.4200	0.2863	0.3132
SONOL	0.1200	0.2150	0.1986	0.1848
STEME	0.3632	0.2711	0.2301	0.0534
VERHE	0.5475	0.2048	0.0319	0.0749
VERPE	0.4067	0.3242	0.1000	0.0440
