# Supplementary material for: Dynamics of Weeds in the Soil Seed Bank: A Hidden Markov Model to Estimate Life History Traits from Standing Plant Time Series
Source: PLoS One. 2015 Oct 1;10(10):e0139278. doi: 10.1371/journal.pone.0139278 (PMC4591344; doi:10.1371/journal.pone.0139278)
Supplement: S3 Table — Yk stands for the probability that the seed bank is in class k at t = 0. Means and standard deviations are reported. These values were obtained using the Gibbs sampler algorithm. (PDF) [file pone.0139278.s007.pdf]

EPPO Code	Y1	Y2	Y3	Y4	Y5	Y6
ALOMY	0.4462+/-0.0422	0.3411+/-0.0598	0.1512+/-0.0454	0.0137+/-0.0121	0.0086+/-0.0083	0.0389+/-0.0122
ANGAR	0.3241+/-0.0424	0.5111+/-0.0851	0.1372+/-0.0689	0.0064+/-0.0038	0.0099+/-0.0082	0.0109+/-0.0047
CHEAL	0.0329+/-0.0012	0.0090+/-0.0034	0.5226+/-0.0186	0.1086+/-0.0188	0.0098+/-0.0121	0.3169+/-0.0284
FUMOF	0.4660+/-0.0823	0.2832+/-0.1458	0.2120+/-0.1127	0.0175+/-0.0107	0.0149+/-0.0103	0.0061+/-0.0046
GALAP	0.1046+/-0.0503	0.1194+/-0.0953	0.4910+/-0.1403	0.1688+/-0.0950	0.0728+/-0.0395	0.0432+/-0.0290
MERAN	0.4742+/-0.0429	0.2752+/-0.0678	0.1891+/-0.0594	0.0201+/-0.0137	0.0070+/-0.0035	0.0341+/-0.0120
PAPRH	0.3418+/-0.0684	0.1817+/-0.1206	0.2562+/-0.1235	0.1732+/-0.1132	0.0177+/-0.0129	0.0292+/-0.0195
POAAN	0.4929+/-0.0403	0.2251+/-0.0421	0.1680+/-0.0371	0.0174+/-0.0115	0.0104+/-0.0091	0.0859+/-0.0206
POLAV	0.2788+/-0.0455	0.0781+/-0.0524	0.4135+/-0.0730	0.0842+/-0.0548	0.0657+/-0.0450	0.0795+/-0.0366
POLCO	0.3303+/-0.0558	0.3072+/-0.0391	0.2404+/-0.0493	0.0171+/-0.0047	0.0137+/-0.0030	0.0910+/-0.0152
SENVU	0.0449+/-0.0418	0.1860+/-0.0921	0.6125+/-0.0881	0.0470+/-0.0183	0.0324+/-0.0238	0.0770+/-0.0328
SINAR	0.4145+/-0.0485	0.4856+/-0.0715	0.0812+/-0.0458	0.0070+/-0.0040	0.0045+/-0.0035	0.0068+/-0.0044
SOLNI	0.2181+/-0.0368	0.0555+/-0.0466	0.5803+/-0.0474	0.0125+/-0.0062	0.0041+/-0.0025	0.1291+/-0.0316
SONAS	0.2529+/-0.0581	0.0515+/-0.0330	0.2978+/-0.1369	0.2680+/-0.1482	0.0988+/-0.0790	0.0306+/-0.0184
SONOL	0.5453+/-0.0649	0.2008+/-0.0719	0.1520+/-0.0973	0.0695+/-0.0547	0.0142+/-0.0063	0.0180+/-0.0078
STEME	0.3944+/-0.0494	0.1675+/-0.0871	0.2934+/-0.0677	0.0492+/-0.0361	0.0137+/-0.0095	0.0814+/-0.0205
VERHE	0.3295+/-0.0492	0.1198+/-0.0773	0.4496+/-0.0572	0.0469+/-0.0390	0.0157+/-0.0107	0.0382+/-0.0136
VERPE	0.3009+/-0.0587	0.1097+/-0.0815	0.4458+/-0.0886	0.1185+/-0.0688	0.0168+/-0.0111	0.0081+/-0.0045
