# Supplementary material for: Dynamics of Weeds in the Soil Seed Bank: A Hidden Markov Model to Estimate Life History Traits from Standing Plant Time Series
Source: PLoS One. 2015 Oct 1;10(10):e0139278. doi: 10.1371/journal.pone.0139278 (PMC4591344; doi:10.1371/journal.pone.0139278)
Supplement: S4 Table — Means and standard deviations of life history trait distributions obtained by the Gibbs sampler algorithm. (PDF) [file pone.0139278.s008.pdf]

EPPO Code	*s* WC	*σ* WC	*φ* WC	*s* OR	*σ* OR	*φ* OR	*s* M	*σ* M	*φ* M	*s* SF	*σ* SF	*φ* SF
ALOMY	0.8303+/-0.134	0.1936+/-0.038	1.2345+/-0.378	0.9180+/-0.047	0.1189+/-0.031	0.4827+/-0.388	0.6994+/-0.144	0.0746+/-0.025	0.4198+/-0.312	0.3063+/-0.183	0.0100+/-0.007	0.5103+/-0.316
ANGAR	0.8902+/-0.083	0.0139+/-0.009	1.1475+/-0.729	0.7120+/-0.135	0.0505+/-0.033	5.5352+/-5.342	0.9320+/-0.037	0.1635+/-0.050	0.1325+/-0.112	0.6394+/-0.225	0.1626+/-0.099	0.3927+/-0.286
CHEAL	0.8815+/-0	0.0039+/-0.001	1.0469+/-0.149	0.5772+/-0.048	0.0069+/-0.001	0.8712+/-1.632	0.8840+/-0.018	0.1568+/-0.004	0.6906+/-0.012	0.6025+/-0.018	0.0662+/-0.003	9.5478+/-4.251
FUMOF	0.9416+/-0.022	0.0464+/-0.019	1.2128+/-0.685	0.6224+/-0.207	0.0159+/-0.011	0.4430+/-0.292	0.5018+/-0.163	0.0159+/-0.009	1.4260+/-1.031	0.6862+/-0.163	0.0048+/-0.003	2.6401+/-1.576
GALAP	0.8761+/-0.075	0.0333+/-0.008	1.4125+/-0.598	0.9220+/-0.056	0.0213+/-0.007	2.9708+/-1.947	0.1079+/-0.043	0.0030+/-0.001	19.754+/-9.492	0.6702+/-0.178	0.0095+/-0.003	3.5396+/-1.511
MERAN	0.9454+/-0.035	0.0098+/-0.002	6.8831+/-4.716	0.6089+/-0.211	0.1569+/-0.035	3.2670+/-2.451	0.8724+/-0.080	0.2322+/-0.093	0.7089+/-0.259	0.6048+/-0.253	0.4056+/-0.192	3.1279+/-1.737
PAPRH	0.8586+/-0.080	0.0479+/-0.022	1.5847+/-0.779	0.4687+/-0.190	0.0254+/-0.013	0.7906+/-0.611	0.3737+/-0.198	0.0003+/-0.000	14.486+/-9.390	0.6828+/-0.170	0.0001+/-9.105	5.3969+/-3.282
POAAN	0.8506+/-0.100	0.1626+/-0.024	0.2907+/-0.219	0.5939+/-0.179	0.0355+/-0.020	0.0523+/-0.037	0.6448+/-0.140	0.0093+/-0.001	7.9713+/-4.492	0.4745+/-0.215	0.0138+/-0.005	1.7188+/-1.561
POLAV	0.8140+/-0.121	0.0199+/-0.005	0.3387+/-0.260	0.5518+/-0.189	0.0016+/-0.001	4.4775+/-2.555	0.7032+/-0.160	0.0523+/-0.015	0.8073+/-0.732	0.3388+/-0.117	0.0475+/-0.011	0.2877+/-0.200
POLCO	0.8737+/-0.065	0.0116+/-0.002	18.615+/-7.266	0.3563+/-0.056	0.0030+/-0.001	15.409+/-3.752	0.9620+/-0.033	0.0844+/-0.018	0.2212+/-0.065	0.8991+/-0.024	0.0276+/-0.031	0.0882+/-0.049
SENVU	0.6817+/-0.184	0.0098+/-0.002	6.7812+/-3.734	0.5457+/-0.241	0.0190+/-0.004	17.145+/-8.910	0.8838+/-0.052	0.0080+/-0.002	15.725+/-4.133	0.8824+/-0.046	0.0281+/-0.011	13.576+/-4.716
SINAR	0.8902+/-0.079	0.0707+/-0.017	2.5662+/-1.081	0.8141+/-0.141	0.1864+/-0.043	1.0109+/-0.508	0.5712+/-0.166	0.0518+/-0.017	0.6594+/-0.400	0.7191+/-0.163	0.1265+/-0.038	4.1502+/-2.150
SOLNI	0.5761+/-0.090	0.0003+/-0.001	3.5725+/-0.457	0.7725+/-0.186	0.0044+/-0.001	181.06+/-144.9	0.8256+/-0.094	0.1173+/-0.012	0.5923+/-0.063	0.8318+/-0.121	0.1305+/-0.011	0.1324+/-0.042
SONAS	0.6301+/-0.145	0.0001+/-0.001	12.776+/-8.903	0.4006+/-0.244	0.0273+/-0.009	23.616+/-12.00	0.9102+/-0.064	0.0144+/-0.004	1.0983+/-0.644	0.3755+/-0.224	0.0215+/-0.007	11.893+/-6.830
SONOL	0.9538+/-0.044	0.0032+/-0.001	30.814+/-16.18	0.3687+/-0.185	0.0058+/-0.003	30.156+/-15.48	0.6805+/-0.154	0.0066+/-0.006	6.5381+/-5.192	0.9247+/-0.032	0.0050+/-0.004	8.0308+/-4.901
STEME	0.8227+/-0.115	0.1166+/-0.014	0.0407+/-0.024	0.7087+/-0.175	0.0676+/-0.019	0.9026+/-0.237	0.8362+/-0.104	0.0164+/-0.004	1.0896+/-0.838	0.3828+/-0.191	0.0063+/-0.003	4.0681+/-3.717
VERHE	0.8838+/-0.087	0.1084+/-0.017	0.6555+/-0.455	0.5632+/-0.200	0.0175+/-0.003	2.2369+/-1.274	0.5149+/-0.166	0.0017+/-0.001	1.1207+/-0.778	0.7211+/-0.165	0.0056+/-0.003	8.0282+/-6.276
VERPE	0.8883+/-0.043	0.0560+/-0.014	0.4799+/-0.408	0.7993+/-0.152	0.0446+/-0.014	3.8367+/-2.598	0.5724+/-0.195	0.0076+/-0.002	5.0286+/-2.598	0.5901+/-0.191	0.0005+/-0.001	8.2883+/-4.958
