# Supplementary material for: Dynamics of Weeds in the Soil Seed Bank: A Hidden Markov Model to Estimate Life History Traits from Standing Plant Time Series
Source: PLoS One. 2015 Oct 1;10(10):e0139278. doi: 10.1371/journal.pone.0139278 (PMC4591344; doi:10.1371/journal.pone.0139278)
Supplement: S5 Table — WC = winter cereals, OR = oilseed rape, M = maize and SF = sunflower. (PDF) [file pone.0139278.s009.pdf]

EPPO Code	WC	OR	M	SF
ALOMY	3.597	13.324	15.291	19.970
ANGAR	50.153	3.334	30.002	6.330
CHEAL	188.130	56.631	6.985	2.000
FUMOF	16.267	54.977	12.654	38.128
GALAP	17.149	14.752	1.540	14.977
MERAN	14.854	2.0185	4.503	1.374
PAPRH	10.698	12.287	33.731	774.850
POAAN	12.647	178.100	7.359	11.133
POLAV	96.170	44.038	12.423	9.500
POLCO	5.257	4.491	43.505	315.170
SENVU	8.696	2.476	7.914	3.654
SINAR	5.590	4.081	10.482	2.320
SOLNI	256.320	2.303	9.536	32.228
SONAS	204.370	1.616	52.652	2.037
SONOL	10.866	2.332	12.469	22.636
STEME	113.040	9.037	39.623	7.477
VERHE	10.641	9.702	134.820	13.326
VERPE	28.097	5.212	10.266	80.301
