# Supplementary material for: Dynamics of Weeds in the Soil Seed Bank: A Hidden Markov Model to Estimate Life History Traits from Standing Plant Time Series
Source: PLoS One. 2015 Oct 1;10(10):e0139278. doi: 10.1371/journal.pone.0139278 (PMC4591344; doi:10.1371/journal.pone.0139278)
Supplement: S1 File — (PDF) [file pone.0139278.s010.pdf]

HMM model on counts

In order to derive a HMM defined on abundance classes of standing weeds and seeds in the bank, we first define a HMM on counts of the same quantities. It is more natural to define dynamics in terms of survival and germination rates, and seed production number from counts. The HMM on classes is then obtained by integration over abundance class ranges.

For one species, a simple model can be written to describe its dynamic between t and $t+1$ given the set of management actions applied during this period. The number Y^{t+1} of seeds at $t+1$ (end of the growing season) is equal to the number Y^t of seeds present at t, the beginning of the growing season, minus the seeds lost either by mortality (Y_m^{t+1}) or by germination (X^{t+1}), and plus the number of seeds produced during the growing season (Y_p^{t+1}):

$$Y^{t+1} = Y^t - X^{t+1} - Y_m^{t+1} + Y_p^{t+1} \qquad \text{(eq. A1)}$$

We assumed that the germination and survival events for one seed follow discrete Bernoulli distributions with respective parameters σ_a and s_a. Thus, the total number of emerged seeds (X^{t+1}) and the total number of dead seeds (Y_m^{t+1}) follow Binomial distributions, respectively, from populations of size Y^t and Y^t- X^{t+1} with probabilities equal to σ_a and 1- s_a.

The number of seeds produced per mature plant fluctuates around the expected seed production number φ_a. In order to take this variability into account, we assumed that the total number of seeds produced (Y_p^{t+1}) follows a Poisson distribution with a mean parameter equal to the number of mature plants (X^{t+1}) multiplied by the seed production per plant φ_a.

Under these assumptions, we can derive two transition probabilities, $P(Y^{t+1}|Y^t,X^{t+1})$ and $P(X^{t+1}|Y^t)$, that define the HMM model on counts. Their evaluation as well as the temporal structure of the HMM model on abundance classes is presented in more detail below.
