# Supplementary material for: Dynamics of Weeds in the Soil Seed Bank: A Hidden Markov Model to Estimate Life History Traits from Standing Plant Time Series
Source: PLoS One. 2015 Oct 1;10(10):e0139278. doi: 10.1371/journal.pone.0139278 (PMC4591344; doi:10.1371/journal.pone.0139278)
Supplement: S2 File — (PDF) [file pone.0139278.s011.pdf]

## HMM model on abundance classes

In practice, information about standing flora is available in terms of abundance classes, not counts. It is also more realistic to work with abundance classes of seeds in the bank. We therefore extended the first HMM model based on counts to a model based on abundance classes. We defined $c_y$ and $c_{x,}$ the abundance classes of seeds and of standing plants observed, respectively. Since no information is available on the distribution of the counts of seeds/emerged plants within a given class, we decided to attach a uniform distribution to each class on the interval defining the class. For the maximal abundance class that is only lower bounded by 101, we attached a geometric distribution of parameter $p = 1/101$ to this class. In our HMM model, the abundance class of mature plants, $c_x^{t+1}$, observed between $t$ and $t+1$, only depends on the abundance class of seeds, $c_y^t$, at the end of the previous year and on $a^t$, the agricultural practices applied between $t$ and $t+1$. The abundance class of seeds, $c_y^{t+1}$, depends on $c_y^t$, the abundance class of seeds, $c_x^{t+1}$, the observed abundance class of plants between $t$ and $t+1$, and $a^t$, the practices applied between $t$ and $t+1$ (Fig. 1 of the main paper).

Hence, the probability that a weed species reaches an abundance class $c_x^{t+1}$ at $t+1$ where $c_y^t$ is its abundance class at $t$, and $a^t$ is the set of agricultural practices applied between $t$ and $t+1$, is:

$$P_{a^t}\left(c_{x^{t+1}} \mid c_{y^t}\right) = \sum_{x^{t+1} \in I_{c_x^{t+1}}} \sum_{y^t \in I_{c_y^t}} P\left(y^t \mid c_{y^t}\right) P_{a^t}\left(x^{t+1} \mid y^t\right) \qquad \text{(eq. A2)}$$

where $Ic_x^{t+1}$ and $Ic_y^t$ represent the intervals of values in abundance classes $c_x^{t+1}$ and $c_y^t$, respectively.

The probability that a weed species population reaches the seed abundance class $c_y^{t+1}$ in the seed bank at $t+1$ given its abundance of seeds $c_y^t$ at $t$ in the seed bank and its abundance of standing plants $c_x^{t+1}$ at $t+1$ where $a^t$ is the set of agricultural practices applied between $t$ and $t+1$, is:

$$P_{a^t}\left(c_{y^{t+1}} \mid c_{x^{t+1}}, c_{y^t}\right) = \sum_{y^{t+1}\in I_{c_{y^{t+1}}}} \sum_{y^t \in I_{c_{y^t}}} \sum_{x^{t+1}\in I_{c_{x^{t+1}}}} \sum_{y_m^{t+1}\in I_{c_{x^{t+1}}}} P_{a^t}\left(y_m^{t+1} \mid y^t, x^{t+1}\right) P\left(y^t \mid c_{y^t}\right) \ldots P\left(x^{t+1} \mid c_{x^{t+1}}\right) P_{a^t}\left(Y_p^{t+1} = y^{t+1} - y^t + x^{t+1} + y_m^{t+1} \mid x^{t+1}\right) \quad \text{(eq. A3)}$$

Transition probabilities were estimated by simulation. In order to compute $P_{a^t}\left(c_{x^{t+1}} \middle| c_{y^t}\right)$ for each of the six classes $c_y^t$, $K$ random realisations of $Y^t$ were generated, following the distribution of $c_y^t$. For each random realisation of $Y^t$, random realisations of $X^{t+1}$ were generated, following the germination rate ($\sigma$) of $a^t$, and attached to the corresponding classes. The same method was used to compute $P_{a^t}\left(c_{y^{t+1}} \middle| c_{x^{t+1}}, c_{y^t}\right)$. The accuracy of this computation can be controlled by the parameter $K$. For small values of $K$, computation is faster but less accurate than with a large value of $K$.

In the abundance class model defined above (eq. A2 and A3), we assumed that abundance classes are defined as intervals of counts, ignoring possible errors in counting. However, this assumption is unrealistic, especially when counts are close to abundance class boundaries. In order to take this potential counting error into account, an error rate ($\varepsilon$) was included in the computation of transition probabilities. It represents the probability of an error of abundance class estimation of +/- one class (for non-extreme abundance classes). This error rate ($\varepsilon$) was fixed to 0.2.
