# Supplementary material for: Dynamics of Weeds in the Soil Seed Bank: A Hidden Markov Model to Estimate Life History Traits from Standing Plant Time Series
Source: PLoS One. 2015 Oct 1;10(10):e0139278. doi: 10.1371/journal.pone.0139278 (PMC4591344; doi:10.1371/journal.pone.0139278)
Supplement: S3 File — (PDF) [file pone.0139278.s012.pdf]

Population dynamics

Species growth rates were estimated from the three LHTs using a Leslie matrix framework that incorporates the LHTs of the weed species into a structured population model (Caswell, 2001):

$$\begin{bmatrix} n_{a_{seed}} \\ n_{a_{plant}} \end{bmatrix}_{t+1} = \begin{bmatrix} s_a(1-\sigma_a)+\sigma_a\varphi_a & 0 \\ \sigma_a & 0 \end{bmatrix} \begin{bmatrix} n_{a_{seed}} \\ n_{a_{plant}} \end{bmatrix}_{t}$$

The asymptotic growth rate λ_a (i.e., when the equilibrium state is reached) is the dominant eigenvalue of the Leslie matrix for management action $a \in A$. We computed the damping ratio $\lambda_a/|\lambda_2|$ (where λ_2 is the subdominant eigenvalue) to measure convergence. The larger this ratio is, the faster the convergence to the asymptotic growth rate will be. Values of the damping ratio per species and per crop type are given in Table S5.
